# Arginine Metabolism in Decidual Macrophages During Pregnancy

**DOI:** 10.1111/aji.70196

**Published:** 2025-12-17

**Authors:** Yonghong Zhang, Jie Mei, Hui Zhang

**Affiliations:** ^1^ Center For Reproductive Medicine and Obstetrics and Gynecology Nanjing Drum Tower Hospital The Affiliated Hospital of Nanjing University Medical School Nanjing China

**Keywords:** Arg‐1, arginine, decidual macrophage, iNOS, metabolism, pregnancy

## Abstract

**Background:**

Maternal immune tolerance to the semi‐allogeneic fetus is essential for successful pregnancy, while immune defense against pathogens must be preserved. Decidual macrophages (DMs) are critical regulators at the maternal–fetal interface, involved in trophoblast invasion, vascular remodeling, and immune modulation.

**Methods:**

This review integrates findings from human studies, animal models, and in vitro experiments to explore how arginine metabolism regulates macrophage polarization and pregnancy outcomes.

**Results:**

Arginine metabolism influences DM function via two major pathways: iNOS promotes M1 polarization and pro‐inflammatory activity, while Arg‐1 supports M2 polarization, tissue remodeling, and immune tolerance. Dysregulation of this balance is associated with pregnancy complications such as pre‐eclampsia and fetal growth restriction. Pathogens like ***Helicobacter pylori*** and ***Mycobacterium tuberculosis*** exploit Arg‐1 activity to evade host immunity. Clinical studies also suggest that L‐arginine supplementation can improve placental function and fetal growth.

**Conclusion:**

Arginine metabolism is a key modulator of macrophage polarization and immune balance in pregnancy. Targeting this pathway may offer novel therapeutic strategies to improve maternal and fetal outcomes.

**Abbreviations**
DM: decidual macrophage
Arg‐1: arginase 1
FGR: fetal growth restriction
IDO: indoleamine 2,3‐dioxygenase
iNOS: induced nitric oxide synthase
interleukin 10: IL‐10
NO: nitric oxide
ODC: ornithine decarboxylase
PD‐L1: programmed cell death ligand 1
PE: pre‐eclampsia
RPL: recurrent pregnancy loss.
TGF: transforming growth factor
Treg: regulatory T

## 1 Introduction

Pregnancy is unique immunological paradigm, requiring maternal immune tolerance toward the semi‐allogeneic fetus bearing paternal antigens, while simultaneously maintaining defense against invading pathogens. To achieve this, the maternal immune system underwent profound adaptations, particularly at the maternal–fetal interface. The maternal–fetal interface is a complex micro‐environment formed by fetal derived trophoblasts, and maternal derived decidual stromal cells and decidual immune cells. In the first trimester of pregnancy, the maternal–fetal interface harbors a diverse repertoire of decidual immune cells, including decidual NK (dNK) cells, macrophages, T cells, DC cells, B cells and NKT cells [1]. In addition to trophoblstss, the placental villi contain other structural and immune cell types [2, 3]. The best‐characterized immune cells resident to the placental villi are yolk sac‐derived macrophages known as Hofbauer cells (HBCs) [4]. HBCs play pivotal roles in maintaining placental homeostasis by regulating angiogenesis and vasculogenesis [5, 6], contributing to tissue remodeling and development [7, 8] and modulating immune response [9]. Moreover, they actively orchestrate defense mechanisms against infections and directly respond to invading pathogens that breach the placenta. The appropriate crosstalk among these cell types underpins successful pregnancy. As the second most abundant immune populations at the maternal–fetal interface, decidual macrophages (DMs) represent 20%–30% of the total immune cells. When properly regulated in phenotype and function, DMs contribute to multiple pivotal events throughout pregnancy, including trophoblast invasion, spiral arterial remodeling and immune tolerance [10, 11]. Dysfunction of DMs—whether in number, phenotype or function—has been implicated in adverse pregnancy outcomes such as spontaneous miscarriage, pre‐eclampsia (PE) and fetal growth restriction (FGR) [12, 13, 14].

Macrophages exhibit significant phenotypic plasticity, responding to environmental cues to polarize along a spectrum ranging from pro‐inflammatory (M1) to anti‐inflammatory (M2) phenotypes [15]. In recent decades, arginine metabolism has emerged as a key regulator of both innate and adaptive immune responses [16]. By modulating arginine metabolism, macrophages can either amplify or suppress immune reactions [16]. Thus, targeting this pathway represents an opportunity for disease modulation through functional remodeling of macrophages. In this review, we explore how arginine metabolism shapes macrophages behavior during pregnancy and contribute to pregnancy‐related complications.

### 1.1 DM at the Maternal–Fetal Interface

DMs are critical for pregnancy success, being involved in a variety of processes, including trophoblast invasion, vascular remodeling and immune tolerance [17, 18]. Based on cytokine profile and functional characteristics, macrophages are classically divided into two subsets: classically activated macrophages (M1 macrophages) and alternatively activated macrophages (M2 macrophages). M1 macrophages are associated with pro‐inflammatory response, with the capacity of presenting antigen and secretion of proinflammatory cytokines. Whereas M2 macrophages have immunosuppressive properties with elevated secretion of interleukin (IL)‐10 and transforming growth factor (TGF)‐β and participate in apoptotic cells clearance and tissue remodeling. DMs exhibit dynamic polarization throughout gestation. During the peri‐implantation period, uterine macrophages predominantly display an M1 phenotype [19]. As extra‐villous trophoblasts invade the uterus, DMs transition to a mixed M1/M2 phenotype, maintaining this balance throughout placentation and the remodeling of spiral arteries [19]. Thereafter, M2 macrophages dominate to maintain maternal‐fetal tolerance until parturition [20], when a transient M1 shift occurs to prepare for labor [13].

DMs engage in complex bidirectional interactions with other cell types. They regulate trophoblast function through cytokines, such as tumor necrosis factor α, CXCL8 and IL‐1β [21, 22, 23]. Conversely, trophoblasts influence DMs via IL‐10, TGF‐β, programmed cell death ligand 1 (PD‐L1), galectin‐9, IL‐34 and receptor activator for nuclear factor‐κB ligand [24, 25, 26, 27]. DMs inhibit Th1 cells via PD‐1/PD‐L1 interaction and modulate decidual T cells through prostaglandin E2 [28, 29] and CD209‐mediated expansion of Foxp3^+^ regulatory T (Treg) cells [30]. Treg cells also promote indoleamine 2,3‐dioxygenase (IDO) production by DMs via the interaction of CD86 and cytotoxic T‐lymphocyte antigen 4 [31]. Interactions with NK cells—abundant in the decidua—are similarly essential, with DMs supporting NK cell function through IL‐15 and TGF‐β secretion and enhancing IDO expression post‐interaction [17, 32].

### 1.2 Macrophage and Arginine Metabolism

Immune metabolism, the study of how nutrients regulate immune function, has highlighted arginine as a pivotal regulator [32]. Arginine serves as a precursor of many molecules [such as ornithine, urea, glutamate, nitric oxide (NO), creatine, proline and polyamines], which play different roles in pregnancy and fetal development [33, 34, 35]. NO is a vasodilator that can regulate flow velocity and blood flow, and is involved in multiple processes associated with pregnancy, including ovulation, implantation, uterine vascular reconstruction and regulation of peripheral vascular resistance [35, 36]. Ornithine and proline are important players for the regulation of gene expression, protein synthesis and angiogenesis [37]. Polyamines are essential for cell growth and viability [38]. Creatine is a key cellular energy metabolite during pregnancy, and appropriate levels of creatine may be crucial for fetal growth and survival [39].

**FIGURE 1:**
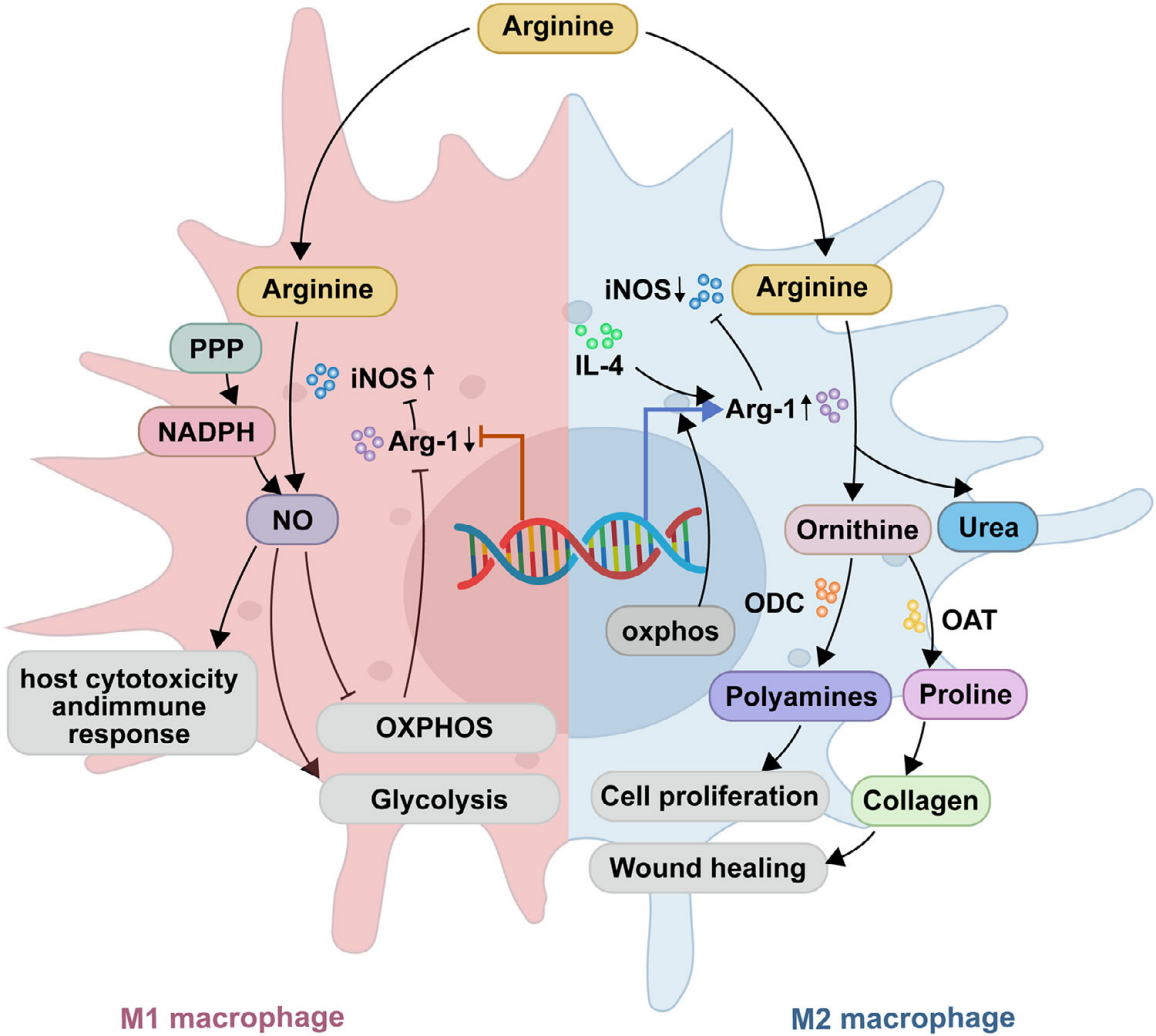
Arginine metabolism modulate macrophage phenotype and function. Arg‐1, arginase 1; DM, decidual macrophage; iNOS, induced nitric oxide synthase.

Two principal enzyme families metabolize arginine: nitric oxide synthase (NOS1‐3) and arginase (arginase 1 and 2). Induced NOS (iNOS), namely, NOS2 is primarily expressed in myeloid cell populations (including monocytes, macrophages, DC and granulocytes) and some T cell subsets [40], while Arg‐1 is found in hepatocytes, macrophages and other myeloid cells and granulocytes [41]. Recently, Arg‐1 has been shown to be expressed by innate lymphoid cells group 2 [42]. At the fundamental level, polarization of M1 and M2 macrophages is achieved through two distinct and antagonistic arginine metabolism pathways mediated by iNOS and Arg‐1. In M1 macrophages, iNOS metabolize arginine into NO, which participates in cytotoxic processes [41] and cell proliferation inhibition [43]. It has been reported that the inhibitory effect of NO on cell proliferation is achieved by suppressing the mitochondrial respiratory pathway [44]. In addition, some studies have shown that NO can inhibit cell proliferation by promoting apoptosis [45], and NO may also indirectly exert antiproliferative effects through interaction with other macrophage cytokines. At the same time, NO can act as a cytokine to regulate cellular physiological functions [46]. In M2 macrophages, arginine is metabolized into ornithine and urea in the presence of Arg‐1. Ornithine is the precursor of polyamines through the ornithine decarboxylase (ODC) pathway and of the proline via the aminotransferase. Polyamines primarily regulate cell proliferation and differentiation, while proline is crucial for collagen synthesis, which is involved in wound healing. Thus, Arg‐1 renders M2 macrophages the capability of tissue remodeling [47] (Figure 1).

The iNOS and Arg‐1 pathways are mutually antagonistic (Figure 1). Compared to M1 macrophages, which rely on glycolysis for energy production [48], M2 macrophages primarily depend on oxidative phosphorylation (OXPHOS) for energy and are associated with M2 macrophage polarization [49]. In M1 macrophages, the pentose phosphate pathway is upregulated as well, leading to increased production of nicotinamide adenine dinucleotide phosphate, which can be utilized for the generation of reactive oxygen species and NO [50]. Excessive NO production with iNOS overexpression leads to host cytotoxicity and immune response in M1 macrophages [51]. Elevated synthesis of NO limit OXPHOS by inhibiting the electron transfer chain, which further inhibited the polarization toward M2 triggered by IL‐4 [52, 53]. Decreased production of NO inhibits the decline of mitochondrial function and promotes polarization of M2 macrophages. On the other hand, upregulation of Arg‐1 is one way by which macrophages limit the availability of arginine and deregulate NO production [54]. This phenomenon became more evident during pathogen infection. *Helicobacter pylori* induces iNOS expression and antimicrobial NO production, but it simultaneously upregulates Arg‐1, leading to ornithine, and ODC, which produces polyamines. Knockdown of ODC by siRNA enhanced *H. pylori* induced iNOS expression and NO release, whereas the addition of spermine abrogated this effect [30]. These findings suggest that *H. pylori* manipulated host immune response by promoting spermine synthesis via the ODC pathway, which in turn suppresses iNOS translation and reduces NO production [55]. Similarly, pathogens exploit Arg‐1 activity in macrophages to limit NO production and evade host immunity. In patients with pulmonary *Mycobacterium tuberculosis*, elevated serum Arg‐1 activity has been reported, particularly in individuals coinfected with helminths. Helminth coinfection drives Arg‐1–expressing type 2 granulomas, exacerbating inflammation and worsening *M. tuberculosis*–associated pathology [56, 57]. Notably, Arg‐1 can also coexist with iNOS. In granulomas, macrophages serve both as anti‐mycobacterial effectors and as host cells for *M. tuberculosis*. In macaques, granulomas exhibit increased expression and enzymatic activity of both iNOS and Arg‐1 compared to non‐granulomatous tissue. Within these granulomas, iNOS activity is enriched in macrophages in the inner regions, while Arg‐1 predominates in the outer layers. This spatial co‐expression of iNOS and Arg‐1 is thought to limit microbicidal activity at the periphery of lung lesions, thereby facilitating tissue repair [58].

### 1.3 Arginine and Pregnancy

A recent systematic review and meta‐analysis by Goto [59] in human studies confirmed the involvement of arginine in placental function and vascular compliance, both of which are crucial determinants of pregnancy outcomes [60]. The analysis also highlighted the beneficial effects of prenatal oral arginine supplementation on birth outcomes. Beyond its established roles, arginine shows promise in pre‐ and peri‐conceptional strategies, offering potential advantages not only for pregnant women but also for their families, healthcare providers, and policy makers. Nevertheless, despite considerable research, many aspects remain insufficiently explored, particularly its influence on the metabolic profile during pregnancy.

Due to the increased demand for arginine by the mother, pregnancy is considered deficiency of arginine. Therefore, whether insufficient maternal arginine supply during pregnancy may lead to adverse pregnancy outcomes has attracted increasing attention.

PE is a pregnancy‐specific syndrome with an incidence of 3%–5%. It is one of the leading causes of maternal, fetal, and neonatal mortality, particularly in low‐ and middle‐income countries [61]. The exact etiology of PE remains unclear, and thus, no definitive preventive measures or treatments are currently available. However, vascular dysfunction leading to impaired placental function is considered a major cause. The NO synthesis pathway is known to play a crucial role in endothelium‐mediated vasodilation, with NO being one of the key factors regulating placental blood flow [62, 63]. Arginine serves not only as the substrate for NO synthesis but also as an oxidative regulator of NO, and its local availability may be critical for endothelial adaptive mechanisms that counteract vasoconstriction in PE. However, comparative studies on maternal arginine levels in normal pregnancy and PE have yielded heterogeneous results. Although some studies report reduced plasma arginine in women with PE [64, 65], others have shown unchanged or even higher levels, particularly in late‐onset PE [66, 67, 68, 69]. Interestingly, Khalil et al. [64] found that the concentration of L‐arginine in maternal plasma decreased during mid pregnancy, while in late stage, no significant difference was found on the concentration of L‐arginine between the control and the pre‐eclamptic group. These studies suggested that it is the development of early‐onset PE rather than late‐onset PE that is associated with alterations in NO metabolism or synthesis. The inconsistency in maternal arginine levels in these studies is mainly caused by differences on sample analysis, different populations and study design [70]. Although the role of arginine metabolism in PE is not fully understood, some studies have attempted L‐arginine as a supplement to prevent PE. For Mexican pregnant women with high‐risk factors for PE, starting arginine supplementation from mid pregnancy until late pregnancy has been shown effectively to reduce the incidence of PE [71, 72]. The mechanism under L‐arginine supplementation might be that L‐arginine alleviated the inhibition of cationic amino acid transporter 1 in PE and increased the synthesis of endothelial NO, improving the vascular reactivity [73].

FGR represents a condition where the estimated fetal weight is lower than expected based on gestational age. FGR increases neonatal morbidity and mortality, and endangers postnatal growth and health. There are multiple hypotheses for the development of FGR. Placental insufficiency is supposed to leading to FGR by reducing the transport of oxygen and nutrients and limiting fetal growth. Arginine promotes the proliferation of embryonic and placental cells by activating the mTOR signaling pathway [74]. Arginine can also regulate placental blood by increasing the synthesis of endothelial NO, promoting the efficiency of material exchange between the placenta and the fetus. Injecting NO synthase inhibitor into mice in the middle and late stages of pregnancy reduce oxygen saturation in the placental labyrinth, border area and decidua area, leading to hypoxia and FGR [75]. However, arginine supplementation promotes fetal growth in FGR rats [76, 77]. Oral administration of NO donor nitroglycerin to pregnant women with FGR ameliorate placental development and promote fetal growth [78]. In late pregnancy (after 33 weeks), continuous intravenous infusion of L‐arginine upregulated the bioavailability of NO as well as fetal weight in FGR [79]. Arginine supplementation at the appropriate time may be crucial for improving fetal development and maternal pregnancy outcomes. Although there have been many studies on arginine supplementation during pregnancy, there is currently no definite mechanism to explain the impact of arginine on pregnant women.

### 1.4 Arginine and Macrophage During Pregnancy

As we have mentioned above, arginine plays an indispensable role in the survival and growth of embryo during pregnancy. Limited researches on arginine metabolism in macrophages at the maternal fetal interface have been done. In early pregnancy, the concentration of arginine in the uterus gradually increases [80]. Arginine feeding to early pregnant mice can significantly improve embryo implantation and reverse the effects of decrease in embryo implantation caused by NOS inhibitors and polyamine synthase inhibitors [80, 81]. Overexpression of iNOS at the implantation site has serious effects, inhibiting iNOS activity in macrophages leading to decreased miscarriage [82]. Since embryo implantation is inflammatory, the invasion of trophoblasts requires the participation of NO and iNOS. Therefore, macrophages at the implantation site are positively correlated with the expression of iNOS and implantation related factors [83]. In our studies, increased expression of decidual iNOS and NO was observed in patients with recurrent pregnancy loss (RPL). High dose of NO donor administration leads to increased embryo loss in vivo [84]. However, Fan et al. [84] found reduced decidual NO levels and downregulated activity of NOS in RPL patients. Although inconsistent, these findings indicate dysregulated NO levels render DMs also dysfunctional, further impairing immune homeostasis at the maternal–fetal interface [84, 85].

During gestation, *Toxoplasma gondii* infection results in multiple complications, such as stillbirths, abortions and congenital malformations. *T. gondii* upregulated iNOS expression in M1 human and mouse DMs. In contrast, Arg‐1 expression was inhibited in DMs with *T. gondii* infection [86]. Therefore, *T. gondii* infection decreased B7‐H4 expression, which further enhances M1 macrophage activation by inducing iNOS expression and hampers immune suppression of M2 macrophages by downregulating Arg‐1 synthesis [86]. After the adoptive transfer of DM from WT mice, the levels of M1‐related functional molecules CD86 and TNF‐alpha were clearly decreased and the levels of the M2‐related functional molecules (CD206, Arg‐1 and IL‐10) were all significantly increased in *T. gondii* infected *B7‐H4^−/−^* mice [86]. The upregulation of B7‐H4 by the adoptive transfer of DM from WT mice could restore the DM dysfunction caused by *T. gondii* infection and eventually alleviate the adverse pregnancy outcome [86]. Thus, arginine metabolism in macrophages is closely associated with pregnancy outcomes even with *T. gondii* infection. Although these studies only provide a basic idea of arginine metabolism modulating DM function, more researches are still needed.

## 2 Conclusion

The arginine metabolism plays a crucial role at the maternal fetal interface. So far, researches on arginine at the maternal–fetal interface have mostly focused on arginine as an essential amino acid and arginine as a precursor for the synthesis of various bioactive substances. There is relatively little research on arginine metabolism in DM regulation. Given the complexity of arginine metabolism, which plays an important role in immunity, metabolism, oxidative stress and angiogenesis, more researches should be done to clarify the specific functions of arginine metabolism in regulating immune responses. Furthermore, whether the arginine metabolism dysfunction leading to the pregnancy complications via changing the immune microenvironment at the maternal–fetal interface is one highly concerning issue in the future.

## Conflicts of Interest

The authors had no conflict to declare.

## Patient/Participant Consent Statement

This study does not involve human participants and therefore does not require patient or participant consent.

## Clinical Study Registration

This study is not a clinical trial and does not require a clinical study registration number.
